# Boundaries of the Origin of Replication: Creation of a pET-28a-Derived Vector with p15A Copy Control Allowing Compatible Coexistence with pET Vectors

**DOI:** 10.1371/journal.pone.0047259

**Published:** 2012-10-22

**Authors:** Sarmitha Sathiamoorthy, Jumi A. Shin

**Affiliations:** 1 Department of Chemistry, University of Toronto, Mississauga, Ontario, Canada; 2 Institute of Biomaterials and Biomedical Engineering, University of Toronto, Toronto, Ontario, Canada; Indian Institute of Science, India

## Abstract

During our studies involving protein-DNA interactions, we constructed plasmid pSAM to fulfill two requirements: 1) to facilitate transfer of cloned sequences from widely used expression vector pET-28a(+), and 2) to provide a vector compatible with pBR322-derived plasmids for use in cells harboring two different plasmids. Vector pSAM is a pET-28a(+)-derived plasmid with the p15A origin of replication (*ori)*; pET-28a(+) contains the pBR322 replicon that is incompatible with other pBR322-derived plasmids. By replacing the original pET-28a(+) replicon–comprising the *ori*, RNAI, RNAII, and Rom–with the p15A replicon, we generated pSAM, which contains the pET-28a(+) multiple cloning site and is now compatible with pBR322-derived vectors. Plasmid copy number was assessed using quantitative PCR: pSAM copy number was maintained at 18±4 copies per cell, consistent with that of other p15A-type vectors. Compatibility with pBR322-derived vectors was tested with pGEX-6p-1 and pSAM, which maintained their copy numbers of 49±10 and 14±4, respectively, when both were present within the same cell. Swapping of the *ori* is a common practice; however, it is vital that all regions of the original replicon be removed. Additional vector pSAMRNAI illustrated that incompatibility remains when portions of the replicon, such as RNAI and/or Rom, are retained; pSAMRNAI, which contains the intact RNAI but not ROM, lowered the copy number of pGEX-6p-1 to 18±2 in doubly transformed cells due to retention of the pET-28a(+)-derived RNAI. Thus, pSAMRNAI is incompatible with vectors controlled by the pBR322 replicon and further demonstrates the need to remove all portions of the original replicon and to quantitatively assess copy number, both individually and in combination, to ensure vector compatibility. To our knowledge, this is the first instance where the nascent vector has been quantitatively assessed for both plasmid copy number and compatibility. New vector pSAM provides ease of transferring sequences from commonly used pET-28a(+) into a vector compatible with the pBR322 family of plasmids. This essential need is currently not filled.

## Introduction

First described by Bolivar and Rodriguez [1], the pBR322 plasmid and its derivatives continue to be among the most widely used cloning vectors in laboratories worldwide (for a review, see ref. [2]. The success of these plasmids has been largely due to early characterization of the molecule, including its nucleotide sequence. One popular pBR322 derivative is the pET vector series, which contains the T7 promoter-driven system first developed by Studier and Moffatt [3]. The T7 promoter allows high expression levels of cloned genes in the presence of T7 polymerase.

Commercial vectors derived from pET vectors are readily available through Novagen, and a commonly used set of derivatives for cloning and expression is the pET-28a-c(+) vectors. As with other pET vectors, expression of cloned sequences is under the control of the T7 promoter and transcription start site. There is also an added level of control offered by the incorporation of the *lac* operator and *lacI* coding sequence [4]. Therefore, expression of a desired coding sequence can be induced using isopropyl ß-D-1-thiogalactopyranoside (IPTG), a lactose analog.

Due to the heavy use of pBR322-derived plasmids–such as the pET, pBAD, pGEM, and pGEX series of vectors–plasmid incompatibility is often a concern when propagating more than one vector within a system. Incompatibility is the inability of two or more vectors to coexist in a host cell stably without external selective pressure (reviewed in ref. [5]). Plasmids derived from a single-parent vector and sharing the same origin of replication (*ori)* belong to the same incompatibility group and cannot stably coexist in one cell (reviewed in ref. [6]). Incompatibility results in highly variable plasmid copy numbers, as well as hereditary instability.

**Figure 1:**
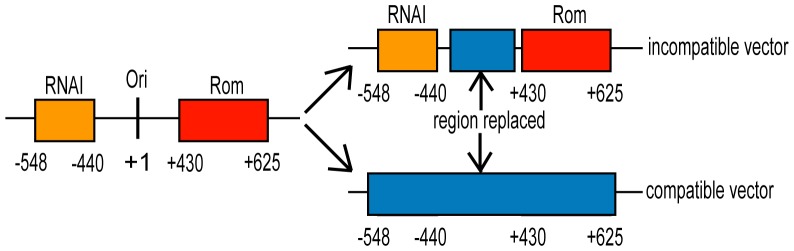
Schematic representation of the replacement of the origin of replication of pBR322 derived vectors. The relative position of RNAI, Rom, and the *ori* are depicted. When RNAI is retained in the nascent vector, vector incompatibility results upon co-transformation with pBR322-derived vectors. Removal of both RNAI and Rom yields a truly compatible vector.

When working with a dual plasmid system, one strategy is to begin with two compatible vectors, such as pBR322 plus p15A, pSC101, or R6K. Another strategy is creation of compatible vectors by exchanging one *ori* with another. The *ori* is the site where replication begins. Elements that control copy number in pBR322-derived vectors include RNAI, RNAII, and Rom (*R*NA *o*ne *m*odulator), and these, with the *ori*, comprise the replicon for the pBR322 family of vectors. RNAI is a transcript of 108 bases that acts as a negative regulator of initiation of plasmid DNA synthesis by sequestering RNAII [7], [8], the primer responsible for initiation of replication [9]. The Rom protein is responsible for strengthening the interaction between RNAI and RNAII and enhances the negative regulatory action of RNAI [10]. The plasmid replicon can therefore be defined as the smallest piece of plasmid DNA that is able to replicate autonomously and maintain normal copy control. Therefore, in order to generate a compatible vector through the exchange of one *ori* for another, the entire original replicon must be removed (Figure 1).

During our work involving assaying protein-DNA interactions in bacterial cells harboring two different plasmids [11], it became necessary to use vectors belonging to different incompatibility groups. One plasmid we use is the pBR322-derived pBAD24. For a compatible plasmid, we chose pACYC, which is derived from p15A: this plasmid uses a completely different mechanism for copy control than that of pBAD24 and pET-28a(+). The majority of our coding sequences were originally cloned into pET-28a(+); to ease the process of transferring the large number of coding sequences, we chose to generate a new plasmid, termed pSAM, that comprises the multiple cloning site of pET-28a(+), but with a p15A *ori*. We therefore replaced the pBR322-derived replicon in pET-28a(+) with the p15A replicon. Due to the roles of RNAI and Rom in plasmid copy control in pBR322-derived vectors, we found it necessary to remove these portions, in addition to the *ori*, in the newly created vector. We demonstrated here that without removal of the full pET-28a(+) replicon, plasmid copy numbers were not maintained in doubly transformed cells, as measured by quantitative PCR.

## Materials and Methods

### Construction of pSAM

Plasmid pSAM was created using the restriction-free cloning strategy described by van den Ent and Lowe [12]. Briefly, the p15A *ori* from pACYCDuet (Novagen, Darmstadt, Germany) was amplified by PCR. The 5′ end of the forward primer had a 22-base overlap with the vector followed by 24 bases of the 5′ end of the p15A *ori*; the 5′ end of the reverse primer had a 21-base overlap with the vector followed by 24 bases of the 3′ end of the *ori*. The reaction was conducted in 1X *PfuUltra* II reaction buffer, 350 µM each of the four dNTPs, 600 nM each primer (pSAMRFFwd and pSAMRFRev, Table 1), 1 µL *PfuUltra* II Fusion HotStart DNA polymerase (Agilent Technologies, Mississauga, ON), 250 ng DNA template, and nuclease-free H_2_O to achieve 50 µL final volume. The thermocycling conditions started with initial denaturation at 95°C, 2 min; 30 cycles of denaturation at 95°C, 20 s; annealing at 55°C, 20 s; extension at 72°C, 30 s; and a final elongation step at 72°C, 3 min. The PCR product was visualized on an agarose gel and purified using the QIAquick Gel Extraction Kit (Qiagen, Toronto, ON).

**Table 1:** Primers used for cloning and qPCR.

Primer name	Sequence 5′-3′	Purpose, product size
pSAMRFFwd	CAAAGGATCTTCTTGAGATCCTGGCTTCTGTTTCTATCAGCTGTCC	Amplification of the p15A *ori*, 1062 bp
pSAMRFRev	CGTGAGCATCCTCTCTCGTTTCGCGGGGCATGACTAACATGAGAA	
*dxs*156Fwd	CGCCGCACCGTCTGTGTCAT	qPCR, 156 bp
*dxs*156Rev	ATGGTTGTTGAGCGCGCCGA	
*bla*136Fwd	ACACCACGATGCCTGCAGCA	qPCR, 136 bp
*bla*136Rev	GCCGAGCGCAGAAGTGGTCC	
*aph*134Fwd	ACTCTGGCGCATCGGGCTTC	qPCR, 134 bp
*aph*134Rev	AACGTCTTGCTCTAGGCCGCG	
*cat*110Fwd	CGGATGAGCATTCATCAGGCGGG	qPCR, 110 bp
*cat*110Rev	AGACCGTTCAGCTGGATATTACGGC	

qPCR  =  quantitative PCR.

bp  =  base pairs.

*ori*  =  origin of replication.

Fwd  =  forward primer.

Rev  =  reverse primer.

The purified product was used as primers for amplification of the pSAM vector. The reaction was carried out in 1X *PfuUltra* II reaction buffer, 200 µM each of the four dNTPs, 400 ng purified PCR product, 100 ng target plasmid pET-28a(+), 1 µL *PfuUltra* II Fusion HotStart DNA polymerase, and nuclease-free H_2_O to achieve 50 µL final volume. The thermocycling conditions started with initial denaturation at 95°C, 3 min; followed by 35 cycles of denaturation at 95°C, 30 s, annealing at 55°C, 1 min, extension at 68°C, 11 min; and a final elongation step at 68°C, 20 min. The product was digested with 1 µL *Dpn*I to degrade the parent vector (New England Biolabs, Pickering, ON) for 3 h, 37°C. Digested product was purified using the QIAquick PCR Purification Kit (Qiagen). Electrocompetent *Escherichia coli* DH5α were transformed with 3 µL purified product. Construction of pSAM was confirmed using restriction endonuclease digestion and DNA sequencing (The Centre for Applied Genomics, The Hospital for Sick Children, Toronto, ON). Plasmids were digested with *Xho*I and *Sac*II (New England Biolabs) to generate two fragments of approximate size 3.1 and 2.0 Kbp.

### Copy Number Determination of pSAM

The copy number of pSAM was determined using quantitative PCR, which offers a reliable and sensitive method for quantifying any sequence of interest in a sample [13]. Absolute copy number was determined using a method similar to that described by Lee et al. [14]. *E. coli* DH5α was transformed with either one or two vectors including pSAM, pACYCDuet, and/or pGEX-6p-1. Therefore, we made five types of cells: three containing individual plasmids pSAM, pGEX-6p-1, or pACYCDuet; one containing both pACYCDuet and pGEX-6p-1; and another containing both pSAM and pGEX-6p-1. The transformation efficiency remained similar for all plasmids tested in single-plasmid transformed cells (within 15% of each other; 3.3×10^6^–2.4×10^7^ CFU/µg DNA). Not unexpectedly, the transformation efficiency for doubly transformed cells was markedly lower, by more than a factor of 100 (2.9×10^4^–6.7×10^4^ CFU/µg DNA). Plasmid pGEX-6p-1 was chosen as a control vector, because it is under the copy control of the pBR322 *ori*, which is similar to that of pET-28a(+), yet contains a different selectable marker from pSAM (pGEX-6p-1 confers resistance to ampicillin, and pSAM confers resistance to kanamycin). This allows for both vectors to be propagated and selected within the same cell. Plasmid pACYCDuet was chosen as a reference for pSAM, as both are under the copy control of the p15A *ori*.

Conducted in triplicate, each culture was grown in selective media overnight. The rate of growth for all transformed cells was similar, with doubling times ranging from 36 to 44 min. After 24 h, the overnight cultures were diluted 500-fold into nonselective media and allowed to grow further for 24 h. The second overnight cultures were diluted 100-fold and grew to an optical density of 0.55–0.65 at 600 nm, corresponding to the exponential phase of growth as determined by a growth curve (data not shown). It has been shown that during the exponential growth phase, plasmid copy number is maximal and can decrease by as much as 80% as the culture ages [15]. Total DNA was extracted from each culture using the QIAamp DNA Mini Kit (Qiagen). RNA-free DNA for the standard curves was generated from untransformed *E. coli* DH5α using the manufacturer’s protocol for the QIAamp DNA Mini Kit. RNA-free plasmid preparations were prepared using the QIAprep Spin Miniprep Kit (Qiagen). RNase digestion was performed with the addition of 20 µg RNase (Fermentas, Burlington, ON) to the re-suspended bacteria before addition of lysis buffer. All DNA was quantified spectrophotometrically (*A*_260_/*A*_280_) on a NanoDrop 2000 spectrophotometer (Thermo Scientific, Mississauga, ON).

Quantitative PCR was performed using the CFX384 real-time PCR detection system and analyzed using CFX Manager™ software version 3.1 (BioRad, Mississauga, ON). Optimal annealing temperature was determined to be 60°C using a temperature gradient from 55–70°C. The reaction mixture contained 1X Express SYBR® GreenER™ qPCR SuperMix Universal (Invitrogen, Burlington, ON), 200 nM each of forward and reverse primers (four specific pairs of primers, Table 1), 3 µL template DNA, and nuclease-free H_2_O to achieve 10 µL total volume. The cycling protocol started with an initial denaturation at 95°C, 3 min; followed by 40 cycles at 95°C, 10 s, and 60°C, 30 s; followed by denaturation at 95°C, 2 min, and re-annealing at 65°C, 5 s. A melt curve analysis was performed to ensure specific amplification of one product. The curve was generated with a melt ramping from 65–95°C, rising 0.5°C each step, with 5 s at each step.

**Figure 2:**
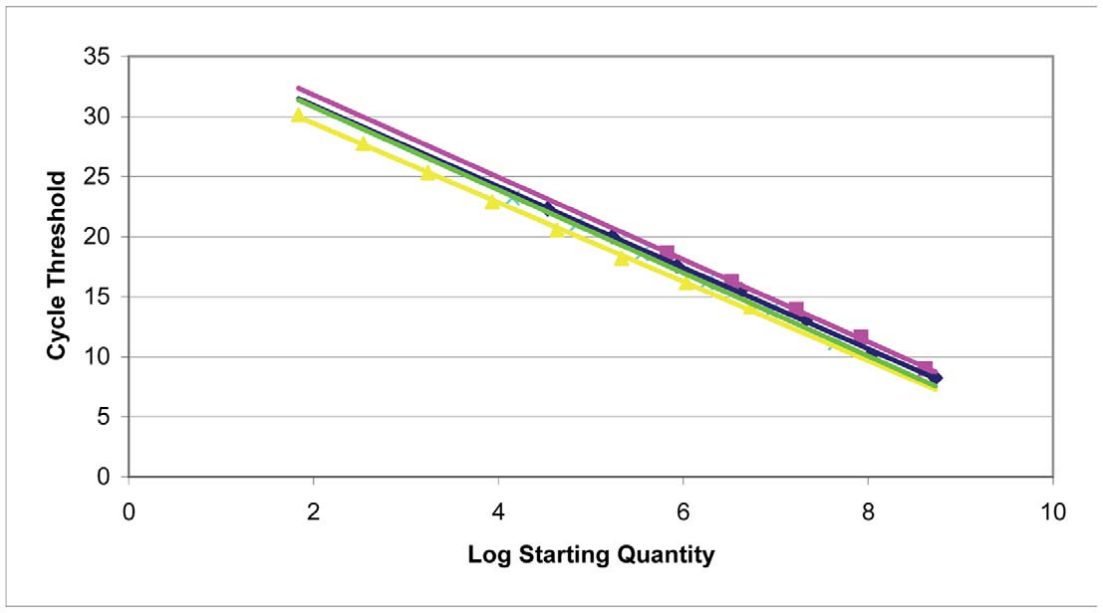
Standard curve used to quantify plasmid copy number in *E. coli.* Plasmid standard curves were generated using known amounts of plasmid DNA. The linear dynamic range was determined to be from 5.37×10^6^ to 69 copies of genomic DNA (yellow), 4.40×10^7^ to 1.41×10^4^ copies of pSAM (green), 4.18×10^8^ to 6.69×10^5^ copies of pACYC (pink), and 5.32×10^8^ to 3.41×10^4^ copies of pGEX-6p-1 (blue). Regression analysis displayed *R* ^2^ values of 0.999 for pSAM, 0.998 for pGEX-6p-1, 0.998 for pACYCDuet, and 0.999 for genomic DNA.

A five-fold serial dilution series of DNA was used to construct the standard curve for the chromosomal gene *dxs*, pSAM gene *aph*, pACYCDuet gene *cat*, and pGEX-6p-1 gene *bla*. The gene *dxs* encodes D-1-deoxyxylulose 5-phosphate synthase and is an essential single-copy gene in *E. coli* [16]. The gene *aph* encodes aminoglycoside phosphotransferase, *bla* encodes ß-lactamase, and *cat* encodes chloramphenicol acetyltransferase [17]–[19]. All reactions had efficiencies between 95.4% and 100.5%, and the standard curves possessed *R* ^2^ values greater than 0.998 from regression analysis (Figure 2). Copy numbers of the plasmids and *dxs* were determined using the following equation:

Mass required for one copy  =  (genome or plasmid size in bp)(1.096×10^−21^ g/bp).

The C_T_ values for standards and samples were measured in triplicate. The exact copy numbers of the targets were determined by interpolating the C_T_ values of the samples from the standard curve. The copy number of the plasmid in each sample was normalized to the copy number of *dxs* to generate the plasmid copy number per cell.

### Transformation Efficiency with Various Plasmids

1.2 ng DNA in 3 µL water was combined with 100 µL chemically competent cells, kept on ice 30 min, followed by heat shock at 42°C for 30 s. Cells were combined with 900 µL SOC broth pre-warmed to 37°C, followed by recovery at 37°C for 2 h with aeration, and cultured on LB agar with appropriate antibiotics. Transformation efficiency was determined by calculating CFU per µg DNA.

### Growth Rate of Transformed DH5α

*E. coli* containing vectors were grown overnight. Overnight cultures were diluted 100-fold and allowed to grow at 37°C with aeration. Samples from the growing cultures were obtained at 15 min intervals, and optical densities were measured by UV/Vis spectrometry (Beckman DU 640). Optical density at 600 nm was plotted against time to generate growth curves and calculate doubling time.

## Results and Discussion

### pSAM was Synthesized with the p15A Origin of Replication

The cloned region of pSAM was sequenced, and insertion of the p15A replicon was confirmed (sequence provided in Figure S1). Similar to pET-28a(+), pSAM contains the T7 promoter and transcription start site, poly-histidine tag coding sequence, T7 tag coding sequence, the same multiple cloning site, T7 terminator, *lacI* coding sequence, *aph* coding sequence, and an f1 origin. Unlike pET-28a(+), pSAM is under the copy control of the p15A replicon.

Although exchange of one *ori* for another has been previously performed, including that by Mayer et al., Finkelstein et al., and Zeng et al. [20]–[22], plasmid copy number has not been assessed using a sensitive method such as quantitative PCR (qPCR), which is a relatively new technique. Traditionally, plasmid copy numbers were assessed using methods including Southern blots [23], agarose gel densitometric quantification of gel bands [24], [25], concentrations of plasmids in relation to cell count [26], and other methods that do not give precise copy numbers. Due to the highly sensitive nature of qPCR, this technique allows accurate measurement of plasmid copy number.

### The Copy Numbers of pSAM and p15A are Consistent

Plasmid pSAM was expected to retain the copy number of 10–50 copies per cell associated with p15A [15], [27], [28]. Copy number was determined by qPCR using total DNA as template. Purification of total DNA provides a DNA sample that contains both genomic and plasmid DNA. Copy number assessment was attempted using crude cell lysates as described by Carapuca et al. [15]; however, amplification efficiency was influenced by cell lysate, and assessment of copy number by optical density and cell count by plating led to highly variable numbers (more detail provided in File S1 and Figure S2).

**Figure 3:**
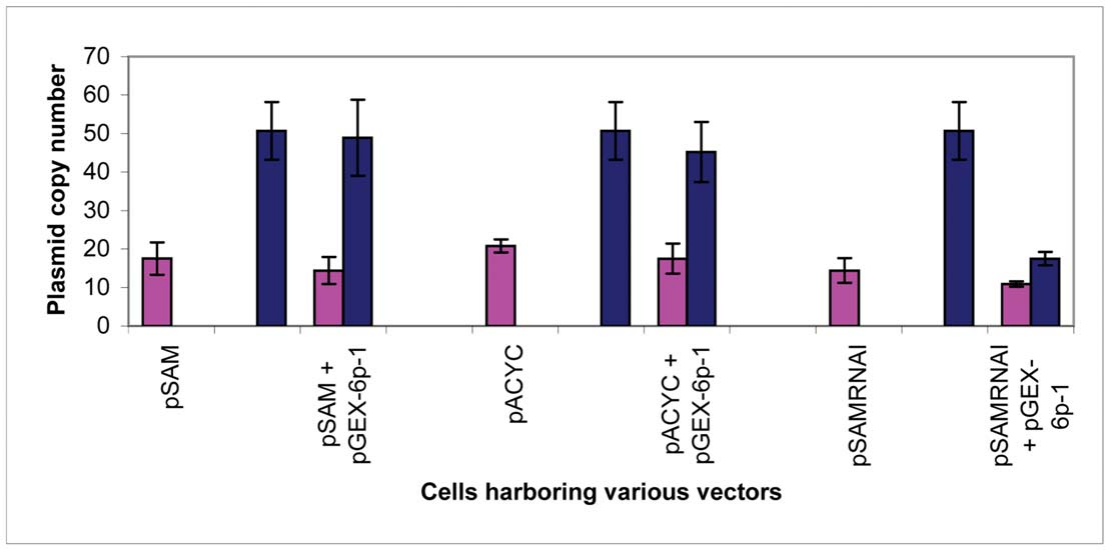
Plasmid copy number per cell as determined by qPCR. Plasmid copy numbers were determined in *E. coli* DH5α harboring one or two vectors. In pink is the copy number of all vectors with the p15A *ori*; in blue is the copy number of pGEX-6p-1, which is controlled by the pBR322 *ori*. Plasmids pSAM and pGEX-6p-1 retained their copy numbers both individually and when cells were doubly transformed. This trend for pSAM was similar to that observed with pACYCDuet (a member of the p15A family) both individually and when doubly transformed with pGEX-6p-1. Plasmid pSAMRNAI causes a decrease in the copy number of pGEX-6p-1 when cells were doubly transformed due to the presence of the pET-28a(+)-derived RNAI retained by pSAMRNAI.

The melt curve analysis ensured primer specificity and amplification of one PCR product. The linear dynamic range of the standard curve was determined to be from 5.37×10^6^ to 69 copies of genomic DNA, 4.40×10^7^ to 1.41×10^4^ copies of pSAM, 4.18×10^8^ to 6.69×10^5^ copies of pACYCDuet, and 5.32×10^8^ to 3.41×10^4^ copies of pGEX-6p-1 (Figure 2). When converted to number of copies per cell transformed with a single plasmid, the copy numbers of pSAM, pACYCDuet, and pGEX-6p-1 individually were 18±4, 21±2, and 51±8, respectively (Figure 3). The copy number of pGEX-6p-1 was anticipated to be approximately 40 copies per cell, based on gel analysis reported by Novagen [29]. This confirms that the copy number of pSAM is consistent with p15A-type vectors, such as pACYCDuet. Copy number is controlled by the replicon; as such, it was expected that pSAM, which contains the p15A replicon, like pACYCDuet, would retain a similar copy number.

When cells were transformed with two different vectors, the copy number of each was maintained: for cells harboring both pSAM and pGEX-6p-1, copy numbers were 14±4 and 49±10, respectively (Figure 3). Cells harboring pACYCDuet and pGEX-6p-1 had copy numbers of 18±4 and 45±8, respectively. These results further illustrate that pSAM is similar to other p15A-type vectors with regard to its copy number control and plasmid compatibility with pBR322-derived vectors.

Interestingly, compatibility is often assumed and rarely assessed when the *ori* is replaced. We found no published literature where plasmid copy number was assessed in a quantitative manner in cells transformed with two presumably compatible vectors. Routinely, cells are co-transformed with the nascent vector and a compatible vector (both of which are selected for by using antibiotics), and expression of cloned proteins is used as an indicator of compatibility [20]–[22]. However, if two plasmids are compatible, they must be able to stably co-exist within a cell under no selective pressure. Also, the yield of cloned proteins is influenced by many factors, only one of which is the plasmid copy number.

It is vital that the plasmids themselves be assessed to determine their absolute copy numbers per cell. Along with determining the copy number of the single nascent vector, the copy numbers of two compatible vectors in doubly transformed cells must also be assessed. Without accurate determination of plasmid copy numbers in cells transformed with two different vectors, it is incorrect to make assumptions about compatibility and maintenance of copy numbers.

### The Importance of Removing the Full Replicon from the Parent Vector

When exchanging one class of *ori* for another, it is critical that the full replicon of the original vector be removed. The replicon is defined as the region containing the *ori,* RNAI, RNAII, and Rom in pBR322-derived plasmids and related ColE1 plasmids. In the case of commercially available pET-28a(+), the sequence provided by the Novagen website marks the pBR322 *ori* as a single nucleotide denoting the replication start site. In contrast, other researchers have used the term “*ori*” differently. For example, for vectors pBAD24 and pGEX-6p-1, Cordingly et al. and Guzman et al. have defined the pBR322 *ori* as regions containing 697 and 619 base pairs, respectively [30], [31]. These regions of the *ori* contain the RNAI sequence and the one-nucleotide *ori* reported for pET-28a(+). In addition, just downstream and outside of their defined *ori* region is the Rom protein coding sequence. This shows ambiguity as to how the *ori* is defined by different investigators.

It is critical that a standard definition of the *ori* be used, and we use the historical definition of the term *ori* to define the replication start site. If other elements, both *cis* and *trans* acting, such as RNAI and Rom, are involved in copy number control, then this entire region should be defined as the replicon.

Often, one or both of these components of the replicon–RNAI and Rom–are left in the engineered vector [20]–[22]. RNAI is *trans* acting, and is the only means of replication control for pBR322 derived plasmids [32]. When RNAI was incorporated into an otherwise compatible vector, its presence was shown to affect plasmid stability in doubly transformed cells; Moser and Campbell illustrated that maintenance of copy number is dependent on the gene dosage of RNAI, and that multiple RNAI copies within the same construct can also cause a decrease in plasmid copy number [33]. In cases where copy numbers are assessed in single-plasmid transformed cells, these numbers will hold true to the copy number associated with the *ori*. However, when the host cell harbors two plasmids, the engineered vector will interfere with the copy number of the second vector, if RNAI and/or Rom remain in the engineered vector. Therefore, assessment of copy numbers from cells harboring the different plasmids separately vs. cells harboring both plasmids is not necessarily the same, and fidelity of copy number is not achieved in single- vs. double-plasmid transformed cells. Typically, assessment of copy number is only performed on the single-plasmid transformed cells, and the assumption is that this copy number will remain constant in the double-plasmid transformed cells.

### RNAI Confers Incompatibility

RNAI is a critical player in plasmid incompatibility and copy number control. To illustrate the importance of removing RNAI from the engineered vector, we tested the copy number of pSAMRNAI with pGEX-6p-1. The RNAI sequence was determined using the sequence published by Tamm and Polisky [8]. Vector pSAMRNAI contains the p15A replicon; however it retains the RNAI sequence found in pET-28a(+) (more detail provided in File S2 and Figure S3). When RNAI is left intact, the copy number of pSAMRNAI is maintained when cells are singly transformed with pSAMRNAI alone (14±3) or doubly transformed with pGEX-6p-1 (11±1, Figure 3). However, in doubly transformed cells, the pGEX-6p-1 copy number is drastically reduced when tested with pSAMRNAI, from 51±8 in single-plasmid transformed cells reduced to 18±2 in the presence of pSAMRNAI.

In comparison with the pBR322 vector family, p15A governs copy number by a different mechanism that relies on several DNA repeats known as iterons (∼20 base pairs each) in the basic replicon to control copy number and confer incompatibility (reviewed in ref. [34]). Despite the different control mechanism used by p15A, the RNAI retained by pSAMRNAI interferes with the copy control of pGEX-6p-1. Chiang and Bremer illustrated that one plasmid may dominate another compatible vector with regards to copy number in doubly transformed cells [32]. This is unlikely to be the case with pSAMRNAI and pGEX-6p-1, because pGEX-6p-1 copy number was maintained when co-transformed with pSAM.

Therefore, the RNAI sequence, which was left intact in pSAMRNAI, dramatically affected the copy number of pGEX-6p-1, due to its *trans-*acting ability. When altering a plasmid’s *ori*, it therefore is critical to transfer the full replicon and to assess proper copy number, both individually and in the presence of the anticipated compatible vector.

### The Need for Properly Engineered Vectors

There are a disproportionate number of commercially available plasmids from the pBR322 family of vectors for both cloning and expression. This creates a problem when investigators require two compatible vectors. Thus, the exchange of one *ori* for another has become a common occurrence.

Vector maps are routinely used to assess the boundaries of the *ori*. However, we find that even within the pBR322 family of vectors the assigned *ori* can differ based on how one defines the *ori*. It is critical that the historical definition of the *ori* as the point where replication begins be maintained. Also, it is recommended that the other members of the replicon–such as RNAI, RNAII, and Rom–be acknowledged when sequences are annotated and vector maps created.

Commonly, when one *ori* is exchanged for another, assumptions are made pertaining to their compatibility. Rarely is compatibility confirmed. Even when the presence of both vectors in doubly transformed cells are assessed, it is through indirect methods, such as the expression of cloned proteins [20]–[22]. However, evaluation of the actual plasmid copy numbers of newly created vectors, both in single- and double-plasmid transformed cells, is necessary to confirm compatibility and stable heritability. Traditionally, copy numbers have been measured by methods including Southern blots, densitometry of gel bands, and plasmid concentrations in relation to cell count [23]–[26]. However, the advent of qPCR is a game-changer: by allowing highly quantitative copy number assessment, qPCR demonstrates the shortcomings in previously used methods for assessing plasmid compatibility. With the advent of qPCR, however, we now have the means for highly quantitative copy number assessment that improves upon previously used methods for assessing plasmid compatibility.

Here we have created two vectors, one of which, pSAMRNAI, confirms the importance of proper plasmid engineering for quantitative work. The other, pSAM, has been shown to be compatible with pBR322-type vectors. This vector possesses a stable copy number, and pSAM does not affect the copy number of another pBR322-derived vector when doubly transformed. By retaining the full multiple cloning site of pET-28a(+), pSAM facilitates the transfer of cloned genes from the heavily used pET-28a(+) into a compatible vector capable co-existence with other pBR322-derived vectors.

## Supporting Information

Figure S1 **Full sequence of the p15A origin of replication (** ***ori)*** **in pSAM.**
(TIF)

Figure S2 **Standard curve used to assess qPCR with and without whole cells.**
(TIF)

Figure S3 **Construction of pSAM. More experimental details regarding**.
(TIF)

File S1 **Copy number assessment using crude cell lysate.**
(DOCX)

File S2 **Construction of pSAMRNAI.**
(DOCX)
