# Supplementary figures and images for: Boundaries of the Origin of Replication: Creation of a pET-28a-Derived Vector with p15A Copy Control Allowing Compatible Coexistence with pET Vectors
Source: PLoS One. 2012 Oct 22;7(10):e47259. doi: 10.1371/journal.pone.0047259 (PMC3478263; doi:10.1371/journal.pone.0047259)

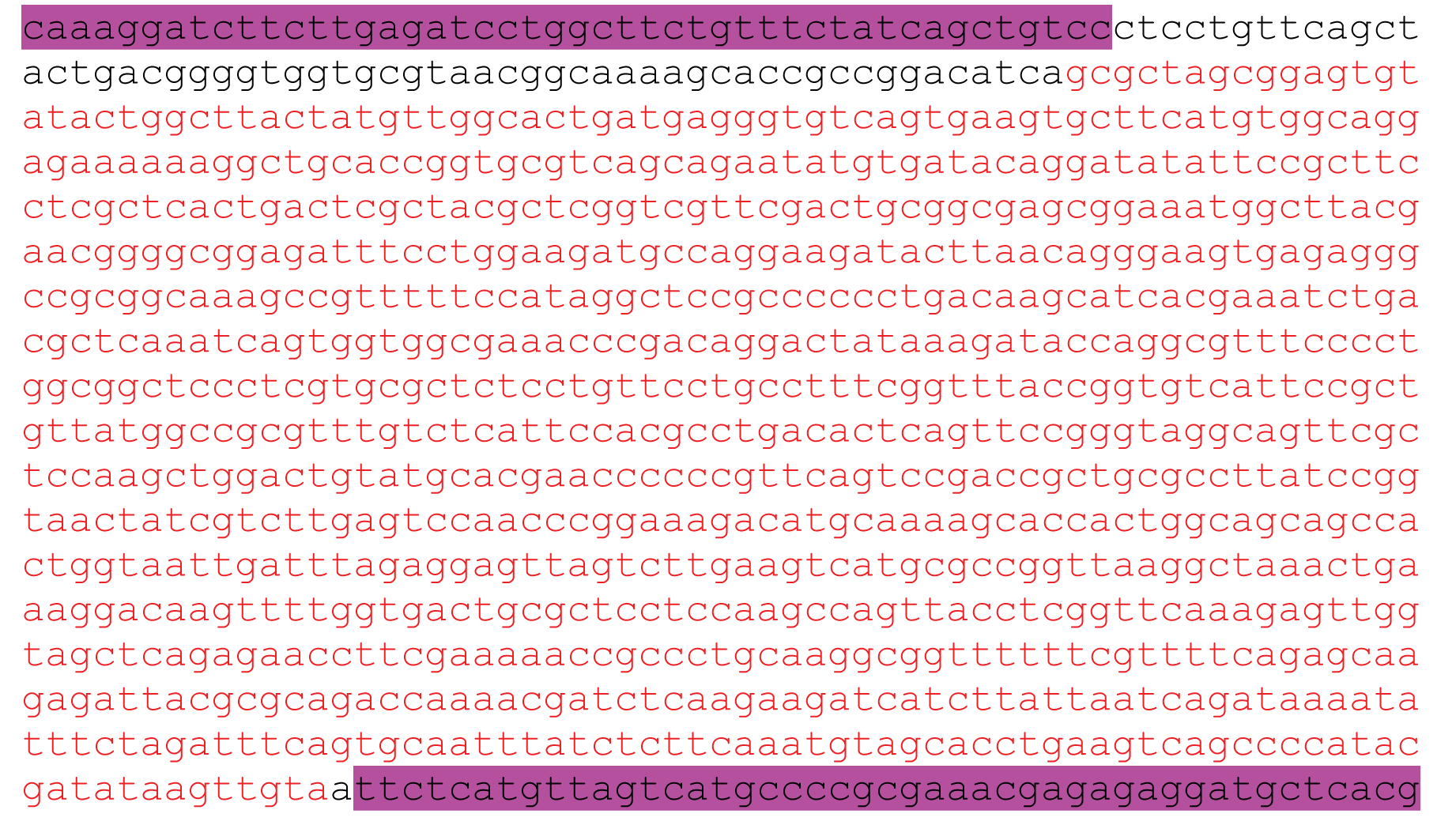

Supplement: Figure S1 — Full sequence of the p15A origin of replication ( ori) in pSAM. (TIF) [file pone.0047259.s001.tif]

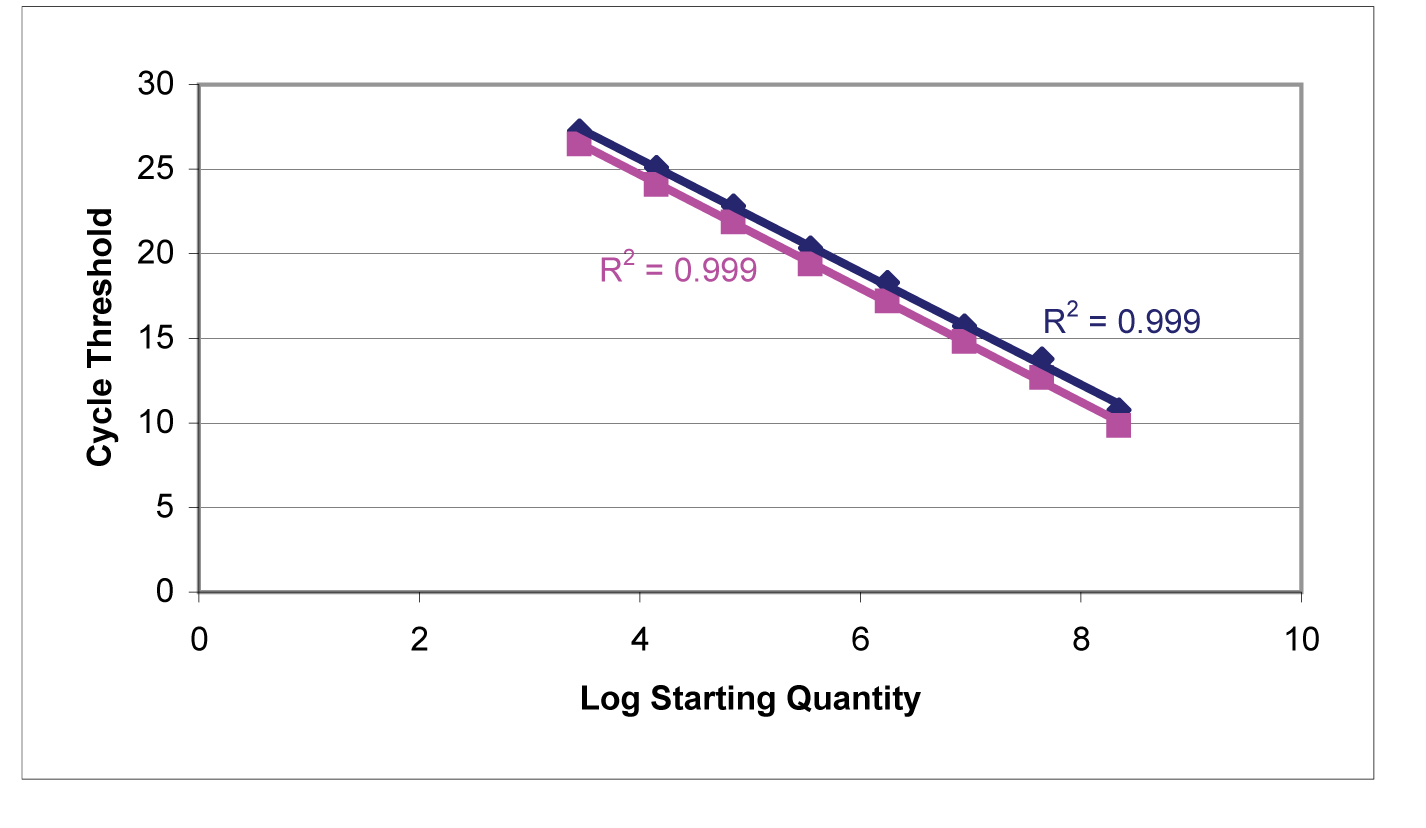

Supplement: Figure S2 — Standard curve used to assess qPCR with and without whole cells. (TIF) [file pone.0047259.s002.tif]

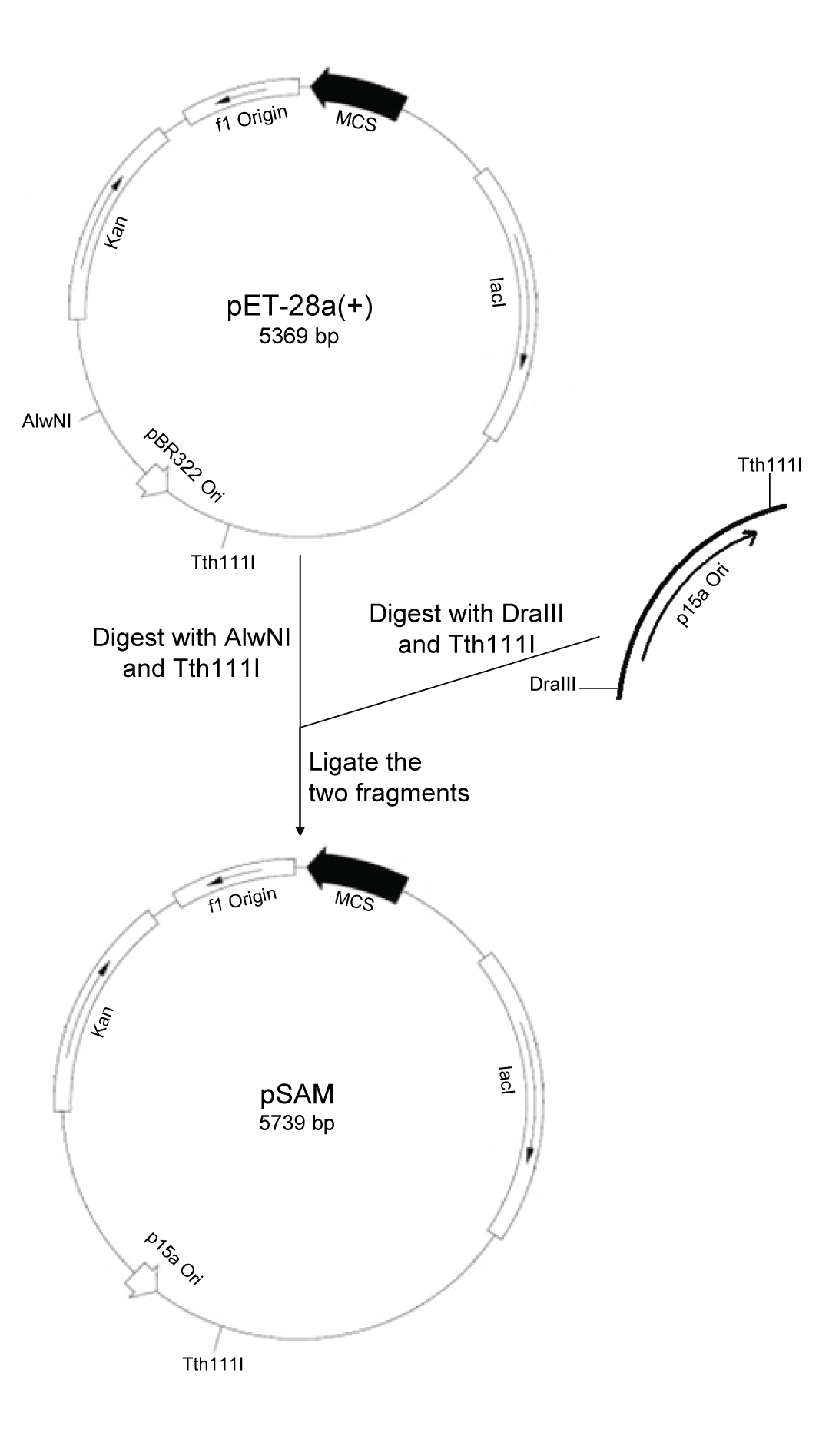

Supplement: Figure S3 — Construction of pSAM. More experimental details regarding. (TIF) [file pone.0047259.s003.tif]
