# Supplementary material for: Boundaries of the Origin of Replication: Creation of a pET-28a-Derived Vector with p15A Copy Control Allowing Compatible Coexistence with pET Vectors
Source: PLoS One. 2012 Oct 22;7(10):e47259. doi: 10.1371/journal.pone.0047259 (PMC3478263; doi:10.1371/journal.pone.0047259)
Supplement: File S1 — Copy number assessment using crude cell lysate. (DOCX) [file pone.0047259.s004.docx]

**Copy number assessment using crude cell lysate**

Plasmid copy number assessment was attempted with crude cell lysate using a method described by Carapuҫa *et al.* (*Mol. Biotechnol*., **2007**, *37*, 120-126). *Escherichia coli* DH5α was transformed with either one or both of vectors pSAM and/or pGEX-6p-1. Therefore, we made three types of cells: one containing pSAM, one containing pGEX-6p-1, and one containing both plasmids. Each culture was grown in selective media overnight, and after 24 h, the overnight cultures were diluted 500-fold into nonselective media and allowed to grow further for 24 h. The second overnight cultures were diluted 100-fold and grew for an additional 4 h.

Quantitative PCR was performed using the method described in the main text, with the exception that template DNA was replaced with 2.0 x 10^5^ – 2.4 x 10^5^ whole cells. The standard curve was generated using known amounts of DNA to which 2.4 x 10^5^ non-transformed *E. coli* DH5α were added. The exact copy numbers of the targets were determined by interpolating the C_T_ values of the samples from the standard curve. The copy number of the plasmid in each sample was divided by the number of cells, as determined by using a correlation between optical density at 600 nm and cell concentration, to generate the plasmid copy number per cell.

The efficiency, as determined by the standard curve using known amounts of DNA spiked with whole cells, was 99.5% to 100%. However, when the same about of DNA was tested using both pure DNA and DNA spiked with whole cells, the C_T_ value was routinely higher for the samples spiked with whole cells (Figure S2). This indicates that whole cells are influencing the PCR reaction due to the likely presence of PCR inhibitors. Plasmid copy number for pSAM was determined to be 9 copies per cell when cells were transformed with the one plasmid. Transformants harboring pGEX-6p-1 alone showed a plasmid copy number of 20. When cells were transformed with both pSAM and pGEX-6p-1, the plasmid copy numbers were 7 and 16, respectively. These numbers were lower than those anticipated for plasmids under the copy control of the origins of replication of either p15A or pBR322.
