# Supplementary material for: Boundaries of the Origin of Replication: Creation of a pET-28a-Derived Vector with p15A Copy Control Allowing Compatible Coexistence with pET Vectors
Source: PLoS One. 2012 Oct 22;7(10):e47259. doi: 10.1371/journal.pone.0047259 (PMC3478263; doi:10.1371/journal.pone.0047259)
Supplement: File S2 — Construction of pSAMRNAI. (DOCX) [file pone.0047259.s005.docx]

**Construction of pSAMRNAI**

Plasmid pSAMRNAI was constructed by PCR amplification of the p15A *ori* from pACYCDuet (Novagen). The primers were designed to incorporate two restriction endonuclease cleavage sites into the PCR product (Figure S2). The reaction was conducted in 1X High Fidelity buffer, 200 μM each of the four dNTPs, 500 nM each of primer p15a-Fwd (5’GGTGCTCCACTGGGTGCTGTTT3’) and primer p15A-Rev (5’CCTTCGACCCAGTCAGCTCCTT3’), 1 μL Phusion high-fidelity DNA polymerase (New England BioLabs), 10 ng template, and nuclease-free H_2_O to achieve 50 μL final volume. The thermocycling conditions started with initial denaturation at 98 ^°^C, 2 min; followed by 30 cycles of denaturation at 98 ^°^C, 10 s, annealing at 63 ^°^C, 20 s, extension at 72 ^°^C, 30 s; and a final elongation step at 72 ^°^C, 7 min. The PCR product was 1060 bp and was examined by agarose gel electrophoresis and digested with *Dra*III and *Tth111*I. Plasmid pET-28a(+) was digested with *AlwN*I and *Tth111*I, followed by treatment with Antarctic Phosphatase (New England BioLabs). Both vector and insert were then ligated to generate pSAMRNAI.
